# Strategies for oocyte collection and *in vitro* embryo production in free-roaming bison herds

**DOI:** 10.1093/conphys/coac058

**Published:** 2022-08-10

**Authors:** Miranda L Zwiefelhofer, Todd Shury, Eric M Zwiefelhofer, Jaswant Singh, Gabriela F Mastromonaco, Gregg P Adams

**Affiliations:** Department of Veterinary Biomedical Sciences, Western College of Veterinary Medicine, University of Saskatchewan, 52 Campus Drive, Saskatoon, Saskatchewan, S7N 5B4, Canada; Department of Veterinary Pathology, Western College of Veterinary Medicine, University of Saskatchewan, 52 Campus Drive, Saskatoon, Saskatchewan, S7N 5B4, Canada; Parks Canada Agency, Government of Canada, 52 Campus Drive, Saskatoon, Saskatchewan, S7N 5B4, Canada; Department of Veterinary Biomedical Sciences, Western College of Veterinary Medicine, University of Saskatchewan, 52 Campus Drive, Saskatoon, Saskatchewan, S7N 5B4, Canada; Department of Veterinary Biomedical Sciences, Western College of Veterinary Medicine, University of Saskatchewan, 52 Campus Drive, Saskatoon, Saskatchewan, S7N 5B4, Canada; Department of Reproductive Sciences, Toronto Zoo, 361A Old Finch Avenue, Toronto, Ontario, M1B 5K7, Canada; Department of Veterinary Biomedical Sciences, Western College of Veterinary Medicine, University of Saskatchewan, 52 Campus Drive, Saskatoon, Saskatchewan, S7N 5B4, Canada

## Abstract

The study was conducted to test the feasibility of protocols for field collection of cumulus–oocyte complexes (COC) for *in vitro* embryo production (IVP) in wild bison. The study was done with captive wood bison during the anovulatory season. In Experiment 1, the efficiency of transvaginal ultrasound-guided COC collection was compared between bison restrained in a squeeze chute without sedation vs in lateral recumbency after chemical immobilization using a dart gun (*n* = 8/group). In Experiment 2, a 2 × 2 design was used to examine the effects of superstimulation treatment [single dose of equine chorionic gonodotrophin (eCG) vs multiple doses of follicle stimulating hormone (FSH)] and method of drug administration (manual injection vs field darting) on COC collection and IVP. In Experiment 1, no difference was detected between chute-restrained vs chemically immobilized groups in the time required to complete COC collections, the number of follicles aspirated (11.5 ± 1.9 vs 9.3 ± 1.8; *P* = 0.4) or the COC recovery rate [COC recovered/follicle aspirated; 58/92 (63%) vs 44/69 (64%); *P* = 0.9]. In Experiment 2, no differences were detected between superstimulation treatments (eCG vs FSH). The total number of follicles available for aspiration did not differ between manual injection and field darting (23.9 ± 2.7 vs 21.6 ± 1.9; *P* = 0.4). Compared with the random start unstimulated group, the embryo production rate was higher [18/132 (14%) vs 53/189 (28%); *P* = 0.04] after wave synchronization and superstimulation. Results suggest that COC collection is equally feasible in a recumbent position after chemical immobilization as those bison restrained in a standing position in a hydraulic chute. Ovarian superstimulation with a single-dose eCG protocol is as effective as a multiple-dose FSH protocol, and field darting is as effective as chute-side administration of superstimulation treatments. The strategies in the present study are ready to be incorporated into field collections in free-roaming bison herds.

## Teaser

Collection of cumulus oocyte complexes in bison using field protocols is feasible. Collections can be done as effectively on sedated-recumbent bison as those restrained in a standing position. Ovarian superstimulation using a single-dose is as effective as a multiple-dose protocol. Field darting is an effective method of administering superstimulation treatments.

## Introduction

A bison genome biobank (repositories of cryopreserved sperm and embryos) is being developed for wood bison (*Bison bison athabascae*), which are classified by the Canadian Government Species at Risk Act (SARA) as Schedule 1 *threatened* (Environment and Climate Change Canada, 2018). The largest and most genetically diverse population of wood bison in the world is located in Wood Buffalo National Park (WBNP), Canada, but is infected by endemic brucellosis and tuberculosis (Joly and Messier, 2005). In 1970, 11 disease-free bison calves were salvaged from a group previously translocated from WBNP to Elk Island National Park (Environment and Climate Change Canada, 2018). These calves were the foundation genetics for a majority of disease-free wood bison conservation herds in the world today (Environment and Climate Change Canada, 2018). The creation and deployment of a bison genome biobank will provide security against future catastrophes (e.g. anthrax outbreak; New *et al.,* 2017), which may result in abrupt population decline and subsequent loss of genetic diversity (Comizzoli *et al.,* 2000). The critically endangered black-footed ferret (*Mustela nigripes*) is a prime example of the deployment a genome biobank for species conservation. Reproductive technologies were used to increase the population of black-footed ferrets from 7 to more than 8000 born in captivity and enabled reintroduction of over 4000 animals back to the wild (Howard *et al.,* 2016).

The biobanking and subsequent redistribution of germplasm collected from isolated free-roaming herds will ensure sufficient genetic diversity for the long-term survival of the species and minimize the risk of disease transmission (Thundathil *et al.,* 2007). In remote locations, helicopters and planes are used to track, monitor and capture wild species such as bison (Caulkett, 2014; Slater, 2020); hence, protocols for the collection of germplasm from bison in wild settings are needed. Cumulus–oocyte complexes (COC) have been collected from bison ovaries recovered post-mortem (Benham *et al.,* 2018) and from live bison restrained in a chute (Cervantes *et al.,* 2017a, 2017b; Palomino *et al.,* 2020; Zwiefelhofer *et al.,* 2022) to create *in vitro* produced embryos, but the collection of COC from immobilized bison has yet to be documented. Current COC collection protocols in bison rely primarily on multiple handlings and ovarian superstimulation treatment (Cervantes *et al.,* 2017a, 2017b; Palomino *et al.,* 2020) to increase follicular size and subsequent COC competence (Vassena *et al.,* 2003). To align with field capture and handling standards to decrease stress in bison (Slater, 2020), reduced handling techniques (maximum of 2 handlings) were investigated in the present study.

As a step towards establishment of a bison genome biobank, two experiments were done to test COC collection protocols designed for use in captive vs wild settings. Experiment 1 was designed to determine the effects of restraint (lateral recumbency after chemical immobilization vs standing position in a hydraulic chute) and allowed us to compare follicular wave status (random vs synchronized) on COC collection efficiency in non-superstimulated bison. Experiment 2 was designed to determine the effects of superstimulation treatment [single dose of equine chorionic gonodotrophin (eCG) vs multiple doses of follicle stimulating hormone (FSH) plus human chorionic gonadotrophin (hCG)] and method of administration (field dart vs manual injection) on COC collection efficiency and IVP.

## Materials and methods

Experiments involved the use of mature female wood bison (*n* = 33), ranging in age from 3–14 years, during the month of July (anovulatory season). The bison were maintained in corrals at the Native Hoofstock Centre near Saskatoon, Saskatchewan, Canada (52°N, 106°W) for the duration of the study and given free access to water and grass hay. Animal use was approved by the University of Saskatchewan’s Animal Research Ethics Board (Protocol No. 20090058) and procedures were done in accordance with the guidelines of the Canadian Council on Animal Care.

### Experiment 1—Effects of restraint and wave synchronization in non-superstimulated bison

**Figure 1:**
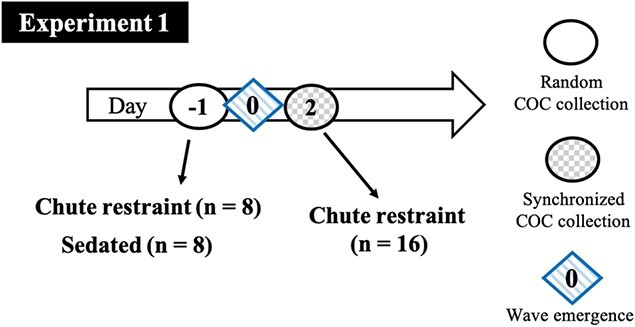
Experimental design for examining the effects of method of restraint and follicular wave synchronization on COC collection in non-superstimulated bison (Experiment 1). Chute restraint (bison collected in standing position without sedation); sedated (bison collected in recumbent position after chemical immobilization); random COC collection (collection at random stage of follicular wave); and n = number of bison per group.

At random stages of ovarian follicular wave development, bison were assigned randomly to two groups (*n* = 8 per group) for transvaginal ultrasound-guided COC collection in either a standing position without sedation (chute restraint) or in lateral recumbency after chemical immobilization (sedated; Fig. 1). The random start collection induced synchronous ovarian follicular wave emergence among bison on the following day (Day 0; Toosi *et al.,* 2013; Palomino *et al.,* 2014), and a second collection was done on Day 2 (synchronized start). The synchronized COC collections were done in the hydraulic chute without sedation (*n* = 16). The experiment was designed principally to examine the effect of restraint method, but also provided an opportunity to examine the effect of random vs synchronized follicular wave status.

**Figure 2:**
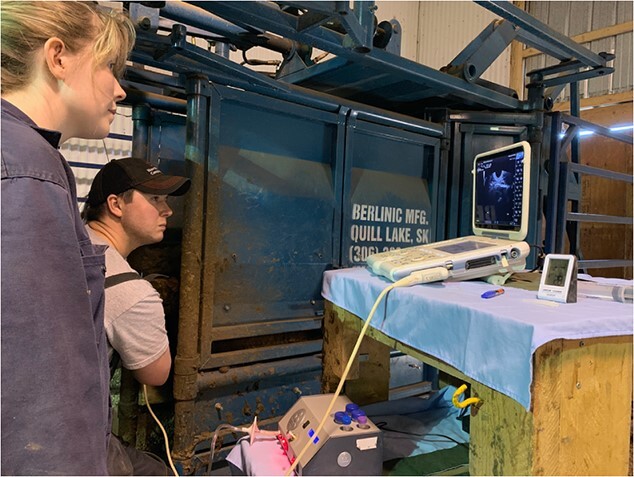
COC collection on bison restrained in a hydraulic chute without sedation during the anovulatory season.

**Figure 3:**
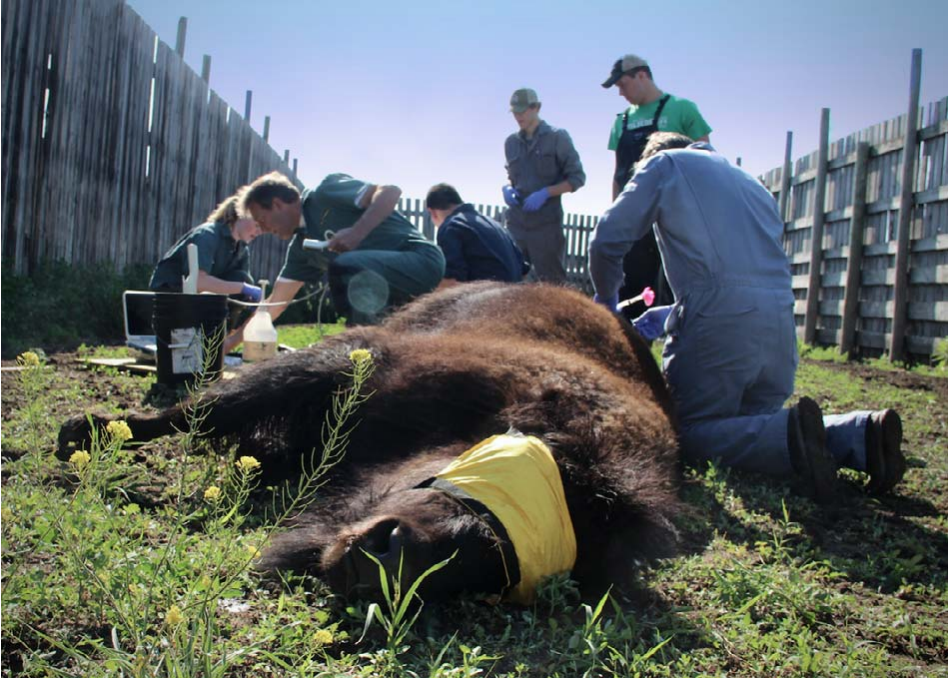
COC collection on a chemically immobilized wood bison during the anovulatory season.

In the chute restraint group, transvaginal ultrasound-guided COC collection was done in the standing position without sedation in a hydraulic chute (Fig 2). Caudal epidural anaesthesia was induced with 5 ml of 2% lidocaine (Lurocaine; Vetoquniol, Lavaltrie, Quebec City, Canada), as previously described (Palomino *et al.,* 2014b). In the sedated group, COC collection was done in lateral recumbency with their left side down, after immobilization using a pre-mixed combination (BAM [butorphanol tartrate (27.3 mg/ml), azaperone tartrate (9.1 mg/ml) and medetomidine hydrochloride (10.9 mg/ml)], 3.5–5.7 ml intramuscularly; Chiron Compounding Pharmacy, Guelph, Ontario, Canada) delivered by a Dan-Inject CO_2_ (Dan-Inject Canada, St. Albert, Alberta, Canada) dart rifle using a 5-ml Dan-Inject 13 mm polycarbonate darts (Fig 3). Sedated bison were given supplemental oxygen intranasally via a portable oxygen cylinder during immobilization if deemed necessary (5–8 l/minute flow) and were blindfolded to reduce stimulation. Sedation was reversed with intramuscular naltrexone hydrochloride (50 mg/ml, 1 cc) and atipamezole hydrochloride (25 mg/ml, twice the volume of BAM administered). Bison were released back to pasture after regaining normal ambulation.

### COC collection and classification

Total bison handling time (duration of time the bison was first touched after capture in chute or full sedation until they were released from the chute or drug reversal was given) and COC collection time (duration of time between insertion and removal of the transvaginal probe) was recorded. The COC collection procedure was done in a manner similar to that previously described (Palomino *et al.,* 2014b). Briefly, the perineum was washed three times with surgical disinfectant, and the ovaries were examined and recorded by transvaginal ultrasonography (MyLab Alpha, Esaote North America Inc, Fishers, IN, USA) for later assessment of follicle numbers and size. COC were collected by transvaginal ultrasound-guided aspiration of follicles ≥3 mm in diameter using a short-beveled 18-ga × 2″ disposable needle (WTA, Cravinhos, Sao Paulo, Brazil) connected to a 50-ml conical tube by autoclaved medical grade Polytetrafluoroethylene (PTFE) micro tubing (internal diameter, 0.047 mm; Catalog # BB311-17, Scientific Commodities, Lake Havasu City, AZ, USA). Follicular contents were aspirated using a regulated vacuum pump set at a flow rate of 12–16 ml/min (BV 003i Digital Vaccum Pump, WTA, Cravinhos, Sao Paulo, Brazil) into collection medium (BO-OPU, Catalog # 51001, IVF Bioscience, Falmouth, Cornwall, UK) maintained at 37°C. The follicular aspirate was filtered through a 75-μm mesh (IVF Oocyte Filter, Partnar Animal Health, Ilderton, Ontario, Canada) and stereomicroscopy (M8; Wild Heerbrugg, Heerbrugg, Switzerland, and SMZ800, Nikon Instruments Inc., NY, USA) was used at 10× magnification to recover the COC. The COC were morphologically classified as described previously (Cervantes *et al.,* 2017b; Zwiefelhofer *et al.,* 2022) and combined into a high-quality group (compact good, compact regular and expanded) and low-quality group (compact poor, denuded and degenerate).

### Experiment 2—Effects of superstimulation treatment and method of administration

**Figure 4:**
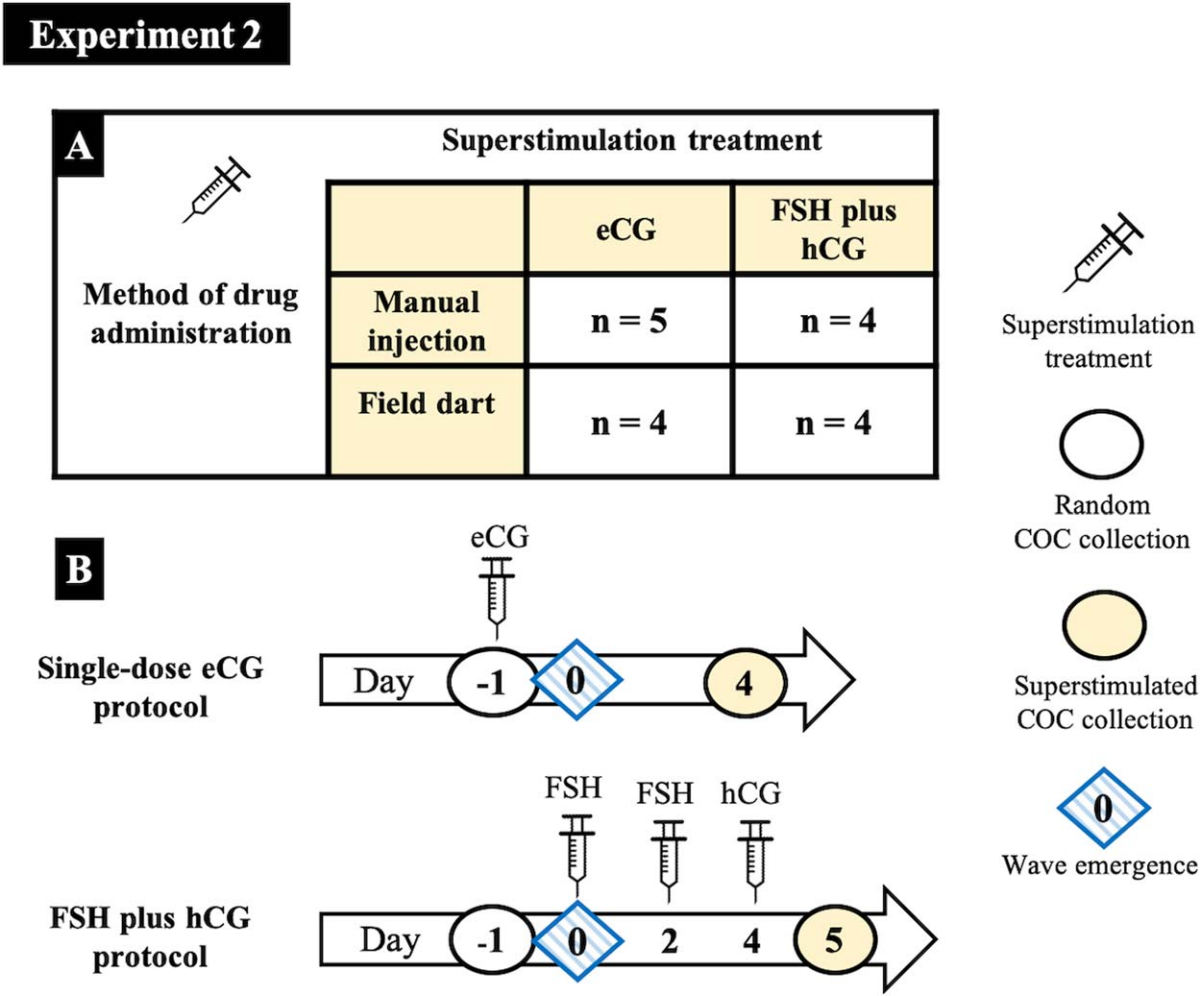
(A) A 2 × 2 experimental design for examining the effects of superstimulation treatment (single dose of eCG vs multiple doses of FSH) and method of administration (field dart vs manual injection) on COC collection efficiency and IVP. (B) Ovarian superstimulation treatment protocols, single-dose eCG (5000 IU equine chorionic gonadotrophin) and FSH plus hCG (two injections of 200 mg FSH and 2000 IU human chorionic gonadotrophin). n, number of bison per group (Experiment 2).

A separate group of bison (*n* = 17) underwent transvaginal ultrasound-guided COC collection in a standing position without sedation, at random stages of the follicular wave. Using a 2 × 2 experimental design, bison were assigned randomly to two groups in which ovarian superstimulation was induced using a single-dose eCG (*n* = 9) vs multiple doses of FSH plus hCG (*n* = 8; Fig. 4). Half of the bison in each superstimulation group were treated by manual intramuscular injection in the chute (*n* = 9) or by field darting (3 cc Type ‘U’ aluminium barrel dart with a 1.25-inch needle and gelatin collar, Pneu-dart, Williamsport, PA, USA; *n* = 8; Fig. 4). Ovarian superstimulation was induced in the eCG group by a single 5000 IU dose of eCG im (Folligon, Merck animal health, Kirkland, Quebec City, Canada) given at the time of the random start collection (Day −1; Day 0 = expected day of follicular wave emergence; Zwiefelhofer *et al.,* 2022). Superstimulation was induced in the FSH group with 200 mg FSH im (Folltropin-V, Vetoquinol NA Inc., Lavaltrie, Quebec City, Canada) on Day 0 and Day 2, and *in vivo* maturation was induced by treatment with 2000 IU hCG im (Chorulon, Merck Animal Health, Summit, NJ, USA) on Day 4 (Cervantes *et al.,* 2016). The second COC collection was done on Day 4 for the eCG group (Zwiefelhofer *et al.,* 2022) and on Day 5 for the FSH group (34 hours after hCG; Cervantes *et al.,* 2017b).

### *In vitro* maturation, fertilization and culture

High- and low-quality COC were processed for embryo production, with the exception of degenerate COC, which were discarded, as described for Experiment 1. The COC were washed and loaded into 1.5-ml sonification tubes (Active Motif, Carlsbad, CA, USA) with 1 mL (BO-HEPES-IVM; Catalog # 71001, IVF Bioscience) into a portable incubator (Lab Mix Portable Incubator, WTA), as previously described (Zwiefelhofer *et al.,* 2022). The COC from the random and eCG collections were matured *in vitro* for 25–28 hours, as described previously (Zwiefelhofer *et al.,* 2022). In the FSH group, COC were collected one day later than in the eCG group to allow *in vivo* maturation (Cervantes *et al.,* 2017b). However, only 20 of 96 COC collected from this group were expanded (matured); therefore, the non-expanded COC (*n* = 76) were matured *in vitro* for 25–28 hours while the 20 expanded COC were matured *in vitro* for a maximum of 4 hours.

The COC (*n* = 5–20 per tube/group) were removed from the maturation tubes and washed once in a prewarmed and equilibrated 500 μl well of bovine IVF medium (BO-IVF, IVF Bioscience, UK). Each group of COC were transferred to a pre-warmed and equilibrated 90 μl drop of BO-IVF covered with oil in a 35-mm petri dish. The dishes were placed in an incubator (Miri Benchtop Multi-room Incubator, Esco Medical ApS, Denmark) at 38.8°C in 5% O_2_, 5% CO_2_ and 90% N_2_. Two 0.5-ml straws of frozen–thawed wood bison semen were thawed and washed twice with 4 ml of 37.0°C BO-SemenPrep (Catalog #71003, IVF Bioscience) as described previously (Zwiefelhofer *et al.,* 2022). Each 90 μl drop of BO-IVF containing COC were fertilized with the prepared semen to a final concentration of 2.0 × 10^6^ sperm/ml. The COC and sperm were co-incubated at 38.8°C in 5% O_2_, 5% CO_2_ and 90% N_2_. Eighteen hours after the start of co-incubation with sperm, presumptive zygotes were denuded in wash medium (WASH; Catalog # 51002, IVF Bioscience) and washed in 500 μl of the pre-warmed and equilibrated in culture medium (BO-IVC, Catalog #71005, IVF Bioscience) at 38.8°C in 5% O_2_, 5% CO_2_ and 90% N_2_. Each group of presumptive zygotes was placed in a pre-warmed and equilibrated 90 μl drop of BO-IVC covered with oil in a 35-mm petri dish and incubated at 38.8°C in 5% O_2_, 5% CO_2_ and 90% N_2_. Presumptive zygotes were evaluated for cleavage (≥2 cell) 3 days after *in vitro* fertilization, and embryo evaluation was conducted by monitoring embryos daily from 7–10 days after fertilization and recording the total number of embryos (morulae, early blastocysts, blastocysts and expanded blastocysts).

### Statistical analyses

The SAS program (Enterprise Guide 4.2; Statistical Analysis System Institute Inc., Cary, NC, USA) was used for statistical analyses. Numerical scale data are represented as mean ± SEM and *P*-values were considered significant if ≤0.05.

In Experiment 1, the effect of the animal restraint system (sedation vs chute) on collection time, the number and size of follicles available for aspiration, the number of follicles aspirated and the number and the quality of COC collected was compared by Student’s *t*-test. The follicle aspiration efficiency (follicles aspirated/follicles available) and COC collection efficiency (COC collected/follicles aspirated) was compared between groups by chi-square. The effect of synchronization (random start vs synchronized start) on the number and size of follicles available for aspiration, the number of follicles aspirated and the number and the quality of COC collected was assessed by the Proc Mixed procedure for repeated measures. Follicle aspiration efficiency (follicles aspirated/follicles available) and COC collection efficiency (COC collected/follicles aspirated) were compared between groups by chi-square.

In Experiment 2, the effects of superstimulation treatment (eCG vs FSH) and method of drug administration (manual injection vs field darting) on the number and size of follicles available for aspiration, the number of follicles aspirated and the number and the quality of COC collected were examined by analysis of variance using a general linear model (GLM) factorial design, and Tukey’s *post-hoc* test was used for multiple comparisons if main effects or interactions were statistically different (*P* ≤ 0.05). Proportional data including follicle aspiration efficiency, COC collection efficiency and cleavage and blastocyst rates (based on the total number of COC used in each treatment group) were examined with the GLIMMIX procedure using binomial distribution and link logit function. Chi-square was used to compare the number of embryos produced per bison. The effect of maturation time in the FSH group (4 hours vs 25–28 hours) on cleavage and embryo rates were compared with the GLIMMIX procedure. Superstimulation treatment and method of administration were then combined to create a superstimulation group to be compared with the collection at random stages of the follicular wave. Analysis of variance using a GLM factorial design was used for the numerical endpoints described above, and Tukey’s *post-hoc* test was used for multiple comparisons. Proportional data were examined with the GLIMMIX procedure using binomial distribution and link logit function and the number of embryos produced per bison was compared using chi-square.

## Results

### Experiment 1—Effects of restraint and wave synchronization in non-superstimulated bison

Transvaginal ultrasound-guided COC collection was carried out successfully and without complication in all bison in each group (Figs 2 and 3). The effects of the bison restraint (sedation vs chute) on procedure efficiency are summarized in Table 1. No differences were detected between restraint systems in total handling time, COC collection time, the size and number follicles available, the number and quality of COC recovered or follicular aspiration and COC collection efficiencies. In the sedated group, the total time from darting to ambulatory recovery was 50 ± 1.3 minutes. All bison recovered normally after immobilization and no mortality or morbidity was observed as a result of chemical immobilization.

**Table 1:** The effect of restraint system (hydraulic chute vs sedated) on handling time and COC collection (mean ± SEM) in wood bison during the anovulatory season (Experiment 1)

	Chute	Sedated
Number of bison	8	8
Total handling time^a^	17.5 ± 3.4	23.5 ± 2.0
COC collection procedure time^b^	6.9 ± 1.0	8.9 ± 1.0
Number of follicles		
3–7.5 mm	13.9 ± 2.4	8.4 ± 1.3
≥8 mm	1.5 ± 0.3	1.4 ± 0.2
Total	15.4 ± 2.4	9.9 ± 1.3
Follicles aspirated	11.5 ± 1.88	8.63 ± 1.5
Follicular aspiration efficiency^c^	92/123 (75%)	69/69 (100%)
COC collection efficiency^d^	58/92 (63%)	44/69 (64%)
COC recovered		
High quality	3.4 ± 0.6	4.0 ± 0.9
Low quality	3.6 ± 1.0	1.6 ± 0.3
Total	7.3 ± 1.2	5.5 ± 0.9

^a^Duration of time (minutes) between first touch in the chute or after sedation until release from the chute or administration of drug reversal.

^b^Duration of time (minutes) between insertion and removal of the COC collection probe.

^c^Follicles aspirated/follicles ≥3 mm available.

^d^COC collected/follicles aspirated.

The effects of the synchronization are summarized in Table 2. No differences were detected in any follicular or COC numerical endpoints between the random and synchronization groups. However, the random group had a greater COC collection efficiency than the synchronization [102/161 (63%) vs 74/151 (49%), Chi-square *P* = 0.02; respectively] but there was no difference between follicular aspiration rates [161/192 (84%) vs 151/191 (79%), *P* = 0.7; respectively].

**Table 2:** The effect of ovarian follicular wave synchronization on COC collection (mean ± SEM) in wood bison during the anovulatory season (Experiment 1)

	Random^a^	Synchronized^b^
Number of bison	16	16
Number of follicles		
3–7.5 mm	12.6 ± 1.5	9.7 ± 1.3
≥8 mm	1.5 ± 0.2	0.9 ± 0.2
Total	12.8 ± 1.6	11.9 ± 1.5
Follicles aspirated	10.1 ± 1.2	9.4 ± 1.2
Follicular aspiration efficiency^c^	161/192 (84%)	151/191 (79%)
COC collection efficiency^d^	102/161 (63%)^e^	74/151 (49%)^e^
COC recovered		
High quality	3.7 ± 0.5	3.1 ± 0.7
Low quality	2.6 ± 0.6	1.6 ± 0.4
Total	6.4 ± 0.8	4.6 ± 0.8

^a^COC collection done at random stages of the follicular wave.

^b^Follicles aspirated/follicles ≥3 mm available.

^c^COC collection done 3 days after random start collection (Day 2 of wave emergence).

^d^COC collected/follicles aspirated.

^e^Within rows, values with no common superscript are different (*P* ≤ 0.05).

### Experiment 2—Effects of superstimulation treatment and method of administration

The effects of superstimulation treatment (eCG vs FSH) and method of drug administration (manual injection vs field darting) on follicle numbers, COC collection and *in vitro* embryo production are provided in Table 3. The superstimulation treatment and method of drug administration had no difference in the total number of ≥3 mm follicles available (*P* = 0.4) or the follicles aspirated (*P* = 0.7). However, the dart administered group had fewer ≥8 mm follicles than the manually injected groups (*P* = 0.04). The eCG group that was administered by darting had the highest follicular aspiration efficiency (method of administration × superstimulation interaction; *P* = 0.03; Table 3) and the lowest COC collection efficiency (method of administration x superstimulation interaction; *P* = 0.001; Table 3). There were a similar number of COC recovered from all groups (*P* = 0.5) and all treatment groups resulted in similar embryo production rates [total = 53/189 (28%)] and embryos produced per bison [total = 53/17 (3.1)]. There was also no difference between the embryo production rate for oocytes collected in the FSH group that were expanded and matured for 4 hours vs non-expanded and matured 25–28 hours [3/20 (15%) vs 23/76 (30%), *P* = 0.2; respectively].

**Table 3:** The effects of superstimulation treatment and method of administration on follicle numbers, COC collection and *in vitro* embryo production (mean ± SEM) in wood bison during the anovulatory season (Experiment 2)

	FSH plus hCG^a^		eCG^b^	
	Manual injection	Dart	Manual injection	Dart
Number of bison	4	4	5	4
Number of follicles				
3–7.5 mm	16.0 ± 6.0	16.3 ± 2.4	12.0 ± 1.5	13.0 ± 2.7
≥8 mm^x^	10.25 ± 1.0	5.5 ± 1.6	10.0 ± 1.3	8.5 ± 1.6
Total	26.3 ± 5.9	21.8 ± 1.6	22.0 ± 2.0	21.5 ± 3.9
Follicles aspirated	23.3 ± 6.2	18.75 ± 3.3	19.0 ± 2.28	21.25 ± 3.99
Follicle asp. Eff.^c^^,^^xy^	93/105 (89%)^s^	75/87 (86%)^s^	95/110 (86%)^s^	85/86 (99%)^t^
COC collect. Eff.^d^^,^^xy^	50/93 (54%)^s^	59/75 (79%)^t^	64/95 (67%)^st^	41/85 (48%)^s^
COC recovered				
High quality	8.3 ± 4.1	10.5 ± 3.0	9.6 ± 2.3	7.0 ± 2.1
Low quality	1.3 ± 0.6	2.3 ± 0.5	0.8 ± 0.8	1.5 ± 0.5
Total	12.5 ± 4.4	14.8 ± 2.8	12.8 ± 2.7	10.3 ± 2.3
Cleavage rate^e^	34/45 (76%)	37/51 (72%)	45/58 (78%)	23/35 (66%)
Embryo rate^f^	13/45 (29%)	13/51 (26%)	15/58 (26%)	12/35 (34%)
Embryos per bison	13/4 (3.3)	13/4 (3.3)	15/5 (3.0)	12/4 (3.0)

^a^200 mg FSH on Days 0 and 2, and 2,000 IU hCG on Day 4 (Day 0 = wave emergence).

^b^5,000 IU eCG on Day –1.

^c^Follicles aspirated/follicles ≥3 mm available.

^d^COC collected/follicles aspirated.

^e^Number of presumptive zygotes cleaved/COC submitted to *in vitro* maturation.

f ^f^Number of morulae and blastocysts produced/COC submitted to *in vitro* maturation.

xy ^xy^Method of administration × superstimulation treatment interaction (*P* ≤ 0.05).

ab ^st^Within rows, values with no common superscript are different (*P* ≤ 0.05).

x ^x^Effect of method of drug administration (*P* ≤ 0.05).

Data from the superstimulation treatment and method of administration groups were combined into a single superstimulation group to compare with random collection prior to superstimulation (Table 4). Although the total number of follicles available did not differ significantly between groups (*P* = 0.4), both the number of ≥8 mm follicles (*P* < 0.004) and the follicular aspiration efficiency (*P* < 0.0001) were greater in the superstimulated group than in the non-stimulated random group (Table 4). The superstimulated group also had higher rates of cleavage (*P* = 0.03) and embryo development (*P* = 0.04) and produced a greater number of embryos than the random non-stimulated group (*P* = 0.01; Table 4).

**Table 4:** Comparison of embryo production after COC collection in non-stimulated bison at random stages of the ovarian follicular wave vs bison given superstimulation treatment after follicular wave synchronization in the anovulatory season (Experiment 2)

	Random, non-stimulated	Synchronized, superstimulated^a^
Number of bison	17	17
Number of follicles		
3–7.5 mm	3.4 ± 6.1	8.6 ± 3.2
≥8 mm	3.4 ± 1.5^x^	8.6 ± 0.8^y^
Total	19.8 ± 3.2	22.8 ± 1.7
Follicles aspirated	15.1 ± 9.7	20.5 ± 7.7
Follicular aspiration efficiency^b^	256/336 (76%)^x^	348/388 (90%)^y^
COC collection effiency^c^	151/336 (45%)	214/348 (61%)
COC recovered		
High quality	6.2 ± 1.7	8.9 ± 1.3
Low quality	2.5 ± 2.5	1.4 ± 1.3
Total	8.9 ± 2.0	12.6 ± 1.5
Cleavage rate^d^	70/132 (53%)^x^	139/189 (74%)^y^
Embryo rate^e^	18/132 (14%)^x^	53/189 (28%)^y^
Embryos per bison	18/17 (1.1)^x^	53/17 (3.1)^y^

a ^a^Superstimulation with either eCG or FSH plus hCG.

b ^b^Follicles aspirated/follicles ≥3 mm available.

c ^c^COC collected/follicles aspirated.

d ^d^Number of presumptive zygotes cleaved/COC submitted to *in vitro* maturation.

e ^e^Number of embryos produced/COC submitted to *in vitro* maturation.

ab ^xy^Within rows, values with no common superscript are different (Student’s paired *t*-test *P* ≤ 0.05).

## Discussion

The ability to collect COC from chemically immobilized bison is an important step for the use of advanced reproductive techniques in wild herds. Results of the present study revealed that the COC collection procedure was neither prolonged nor compromised while the bison was in lateral recumbency after chemical immobilization. The overall handling time for COC collection procedures did not warrant supplementary sedation for any individual. Animal captures in the wild involve additional time for induction and recovery from sedation in chemically immobilized bison, which was not recorded in this study. However, the COC collection time recorded in the present study was within typical handling times for sedated captures for standard wildlife conservation activities (i.e. 20 minutes; Slater, 2020). Furthermore, the COC collection procedure may be done concurrent with the activities of others related to the collection of biological data, samples for disease screening and securing tracking collars (Rhyan *et al.,* 2009; Didkowska *et al.,* 2021).

The COC collection efficiencies of the chemically immobilized bison were similar to collections done in a hydraulic chute without sedation. Challenging aspects of COC collection in chemically immobilized bison include down time between individuals to allow for sedation and reversal, the position of recumbency after sedation and the requirement of specialized personnel and supplementary oxygen. Sternal recumbency was not ideal as the perineal region was tilted towards the ground making the angle of palpation and transvaginal approach awkward. In the present study, those performing COC collections preferred the bison to be in left lateral recumbency as they palpate transrectally with their left hand. Rolling the bison from one side to the other, and adjustment to avoid obstructions, was accomplished relatively easily with two or three people. A similar model has been established for oocyte collection in the rhinoceros as several sub-species of rhinoceros are critically endangered, including the northern white rhinoceros (*Ceratotherium simum cottoni*). Although rhinoceros can be acclimated to tolerate serial transrectal ultrasonography of the reproductive tract while standing (Pennington *et al.,* 2019), successful oocyte collection has only been described using transrectal ultrasound-guided follicular aspiration while in lateral recumbency after chemical immobilization (Hermes *et al.,* 2009).

The goal of synchronizing ovarian follicular wave emergence is to schedule COC collection at a time when most of the follicles ≥3 mm are in the growing phase (i.e. minimize the number of follicles in static and regressing phases) and take advantage of a greater proportion of COC with developmental competence. Consistent with this expectation, cleavage and embryo development rates were greater in the synchronized group than in the random group in our previous study (Zwiefelhofer *et al.,* 2022). As in the previous study (Zwiefelhofer *et al.,* 2022), the number of follicles available and the number and morphologic grades of COC recovered were similar between the random and synchronized groups in the present study; however, the effect on embryo development was not tested in the present study. In cattle, COC collected on Day 2 of the follicular wave had lower embryo development rates than Day 5 (Vassena *et al.,* 2003). Future studies are required to assess the developmental competence of follicles early in the follicular wave in bison.

In the previous study, the minimal-handling single-dose eCG superstimulation protocol was effective for embryo production in bison (Zwiefelhofer *et al.,* 2022). Prior to this, the most successful superstimulation protocol in bison was a multiple-dose FSH plus hCG protocol (five handlings; Cervantes *et al.,* 2017b). In addition, the intended *in vivo* maturation effect of hCG (Cervantes *et al.,* 2017b) was modest in the present study. The COC expansion rate in the FSH + hCG group was lower than previously reported in Cervantes (2017b) [20/109 (18.3%) vs 92/148 (62.2%), respectively] and resulted in the unanticipated need for *in vitro* maturation of 76 non-expanded COC in the FSH + hCG group. The ineffectiveness of including *in vivo* maturation in the present study may have resulted from the use of a smaller dose of hCG than that used previously (i.e. 2000 IU hCG vs 2500 IU) and/or by diluting the FSH in saline rather than hyaluronan. Interestingly, there was no difference in the rate of embryo development between expanded COC that did not undergo *in vitro* maturation and non-expanded COC that underwent 25–28 hours of *in vitro* maturation in the present study. This emphasizes the importance of expansion of the cumulus cells from the oocyte as an indicator for maturation and readiness of the oocyte for *in vitro* fertilization (Merton *et al.,* 2003).

Whether in captive or free-roaming conditions, bison maintain their wild character and demeanour and are more difficult to handle than cattle. The single-dose, two-time handling protocol provides an alternative to the multiple-dose FSH, five-time handling protocol, and thereby reduces unwanted stress in wild bison (Caven *et al.,* 2022). The elimination of three handlings is an important step in applying these technologies to bison in an effort to minimize damage to themselves, other bison and the handling equipment. In free-roaming herds, the two-time handling protocol may be feasible for COC collections done on bison sedated from a helicopter, with the assistance of a tracking collar to relocate the animal for the second collection. While dart-administered treatment resulted in fewer follicles ≥8 mm than the manual treatment, there was no difference in the total number follicles available, and no effect on the number of COC recovered or embryos produced. Proper dart placement is important for ensuring drug effectiveness and rapid absorption (Larter *et al.,* 2000) and may be mitigated for ovarian superstimulation by increasing the dose of the superstimulation drug given.

Importantly, results of the present study document that COC collection at a random stage of the follicular wave is feasible and may be appropriate in the field. Results also showed, however, that synchronized, superstimulated COC collections resulted in a greater number of large follicles, COC recovered and embryos produced than random collections. Using the embryo production rates reported herein for random and synchronized-superstimulated collections (1.1 and 3.1 embryos per bison, respectively), for example, two captures of the same five bison 5 days apart (10 captures) would result in the production of 21 embryos [(1.1 × 5) + (3.1 × 5)]. To produce the same number of embryos using single random collection, 19 bison would need to be collected (19 captures). While single random collection may be more costly, it would provide greater genetic diversity of germplasm. Embryo production from a single random COC collection may also be expected to be more variable since follicles at different stages contain COC of varying competence (Merton *et al.,* 2003; Vassena *et al.,* 2003). The latter is reflected in a comparison of the results of our previous study (Zwiefelhofer *et al.,* 2022) and the present study; embryo production per COC collected was similar in the superstimulation groups but was 6% vs 14% in the random collection groups in respective studies.

The authors once again recognize that this research was conducted on captive bison in a controlled environment. It is acknowledged that bison in free-roaming conditions will be affected by countless additional variables. However, the promising results from this small foundational study have created the first steps in bridging the gap between embryo technologies in captive bovids and the application of these technologies for conservation purposes.

Progress has been made on transitioning COC collection protocols for use in bison in free-roaming field conditions. In summary, COC collections can be done as effectively on sedated recumbent bison as those restrained in a standing position in a hydraulic chute, and ovarian superstimulation using a single-dose protocol is as effective as a multiple-dose protocol. Finally, field darting is an effective method of administering superstimulation treatments and ovarian superstimulation improves *in vitro* embryo production. Future studies will involve implementation of these techniques for COC collection in free-roaming wild bison herds across North America.

## Funding

This work was supported by Natural Sciences and Engineering Research Council of Canada [RGPIN-2018-04126].

## Authors’ contributions

M.L.Z.: conceptualization, methodology, formal analysis, investigation, data curation, writing-original draft, visualization. T.S.: conceptualization, methodology, investigation, resources, writing-review and editing. E.M.Z.: conceptualization, methodology, investigation, writing-review and editing. J.S.: conceptualization, methodology, resources, writing-review and editing. G.F.M.: conceptualization, writing-review and editing. G.P.A.: conceptualization, methodology, resources, writing-review and editing, supervision, funding acquisition.
